# Ventilation

**Published:** 1855-12

**Authors:** 


### VENTILATION.

Don't let the reader be astounded out of his propriety and declare us insane, because we tell him, it is more important to sleep with a window up in mid winter, than in summer-time. How few people have the gift of thinking! how many have the gift of gab, in the inverse ratio! The less a man thinks, the more he can talk; that is the very reason why our household divinities can discourse indefinitely, ad infinitum, and the other side of it. Whoever heard of a man taking cold who slept in *all out doors?* Well, if sleeping in all out doors, does not give a man a cold, how can sleeping in a part of all out doors give him a cold? Is not that conclusive? Surely none of the unthinking multitude could ask for a more convincing argument than this. But as only the thinking few take a journal like this, we will give a mere hint of an argument, with the carrying out of which, they may amuse themselves in a leisure hour. Pure carbonic acid gas is deadly, it kills in five minutes. In sleeping we breathe out this gas, and a close room confines it; warmth makes it rise to the ceiling, cold condenses and keeps it near the floor. *Verb. sat.*

